# Hepatic steatosis in HIV-HCV coinfected patients receiving antiretroviral therapy is associated with HCV-related factors but not antiretrovirals

**DOI:** 10.1186/gb-2012-13-3-1234

**Published:** 2013-01-15

**Authors:** Valérie Martinez, Thi Dieu Ngan TA, Zahra Mokhtari, Marguerite Guiguet, Patrick Miailhes, Marc-Antoine Valantin, Fréderic Charlotte, Philippe Bertheau, Jean-Michel Molina, Christine Katlama, Eric Caumes

**Keywords:** Open Access, Base Case, Business Model, Long Term Evolution, Hepatic Steatosis

## Abstract

In this paper we evaluate the economic gains of a joint deployment of femtocells and macrocells for the provision of Long Term Evolution (LTE) mobile broadband services in urban environments. Frequency bands of 2.6 GHz and 900 MHz are analyzed and different parameters related to the business model are considered for a 30% market share operator. Results show important benefits for the base case where the service is offered to fixed-broadband clients, up to 75%, for small bandwidths. It results feasible to attribute subscriber loop costs to the radio access network (RAN) costs, so that the service could be offered to non fixed-broadband clients, in both cases of closed access and open access femtocells. However, initial savings result notably reduced, up to 50% less than in the base case if closed access is adopted and up to 13% less for open access. Site reuse reduces the initial savings only in 3%.

## Ipilimumab efficacy: Optimal dose and schedule

